# Challenges facing the veterinary profession in Ireland: 3. emergency and casualty slaughter certification

**DOI:** 10.1186/s13620-017-0102-0

**Published:** 2017-08-04

**Authors:** Manuel Magalhães-Sant’Ana, Simon J. More, David B. Morton, Alison J. Hanlon

**Affiliations:** 1 Escola Universitária Vasco da Gama, Av. José R. Sousa Fernandes, Campus Universitário - Bloco B, 3020-210 Coimbra, Portugal; 2 School of Veterinary Medicine, University College Dublin, Dublin, Ireland; 3 Centre for Veterinary Epidemiology and Risk Analysis, University College Dublin, Dublin, Ireland; 4 School of BioSciences, University of Birmingham, B15 2TT, Birmingham, UK

**Keywords:** Animal welfare, Casualty slaughter, Certification, Emergency slaughter, Focus group, Ireland, Veterinary ethics, Veterinary profession

## Abstract

**Background:**

Veterinarians are faced with significant conflicts of interest when issuing certificates for the transport and slaughter of acutely injured and casualty livestock. In a recent Policy Delphi study, emergency and casualty slaughter certification was a key concern identified by veterinary professionals in Ireland. In this case study (the third in a series of three resulting from a research workshop exploring challenges facing the veterinary profession in Ireland; the other two case studies investigate clinical veterinary services and the on-farm use of veterinary antimicrobials), we aim to provide a value-based reflection on the constraints and opportunities for best practice in emergency and casualty slaughter certification in Ireland.

**Results:**

Using a qualitative focus group approach, this study gathered evidence from relevant stakeholders, namely a representative from the regulatory body, local authority veterinarians with research experience in emergency slaughter, an animal welfare research scientist, official veterinarians from the competent authority, a private veterinary practitioner, and a member of a farming organisation. Results revealed a conflict between the responsibility of private veterinary practitioners (PVPs) to safeguard the welfare of acutely injured bovines on-farm and the client’s commercial concerns. As a consequence, some PVPs may feel under pressure to certify, for example, an acutely injured animal for casualty slaughter instead of recommending either on-farm emergency slaughter or disposal by the knackery service. Among Official Veterinarians, there are concerns about the pressure within processing plants to accept acutely injured livestock as casualty animals. Confusion pertaining to legislation and definition of fitness to travel also contribute to these dilemmas.

**Conclusions:**

Conflicts of interest arise due to the gap between governance and provision to facilitate on-farm emergency slaughter of livestock. Increased availability and acceptance of on-farm emergency slaughter by Food Business Operators (FBOs) would mitigate the need to certify acutely injured animals fit for transport and slaughter and thereby safeguard animal welfare. In the absence of nationwide availability and acceptance of on-farm emergency slaughter by FBOs, consideration should be given to methods to encourage all those involved in the food chain to prioritise animal welfare when in conflict with the commercial value of the animal. Training and guidelines for PVPs on the regulatory landscape and ethical decision-making should become available. The reintroduction of the fallen animal scheme should be considered to support farm animal welfare.

## Background

According to the Farm Animal Welfare Advisory Council (FAWAC), emergency slaughter refers to the on-farm slaughter “*of an otherwise healthy animal which has suffered an injury that prevented its transport to the slaughterhouse for welfare reasons*” [1]. Casualty slaughter, on the other hand, is “*the slaughter at a slaughterhouse, of an injured animal that has been deemed fit for transport under veterinary certification*” [2]. Emergency slaughter of livestock relates mainly to bovines that have suffered an accident and sustained injuries that cause acute pain (e.g. fractures), whereas casualty slaughter usually refers to animals suffering from chronic painful conditions (e.g. lameness) [3].

When issuing certificates for emergency and casualty slaughter (ECS) of bovines, veterinarians are required to meet a number of guidelines and norms [2]. A decision tree for managing acutely injured livestock on farm has been provided by FAWAC [1], but there is no agreement among private veterinary practitioners (PVPs) on the circumstances where an acutely injured bovine animal should be transported [2]. Regulatory provisions require that ECS livestock should either a) be slaughtered immediately on farm (on farm emergency slaughter, OFES) to avoid unnecessary suffering, or b) be transported for slaughter provided the PVP is of the opinion that transport is not likely to cause further injury or unnecessary suffering for the animal (Article 28 of S.I. No. 311 of 2010) [4]. In both cases, the carcase or the live animal must be accompanied to the slaughterhouse by a Veterinary Certificate, issued by the PVP [5]. In addition, according to European legislation, all necessary arrangements should be made in advance to minimise the length of the journey and meet animals’ needs during the journey (Article 3 of EC Regulation No 1/2005) [6]. However, on-farm emergency slaughter is not widely available in the Republic of Ireland; for example, only 4% of abattoirs provided such a service between 2011 and 2013 [2].

During a systematic review of veterinary codes of practice in Europe (including the Veterinary Council of Ireland Code of Professional Conduct), certification emerged as one of chief duties towards society held by veterinarians [7]. Further, in a recent Policy Delphi study, emergency and casualty slaughter certification was a key concern identified by veterinary professionals in Ireland [8]. Veterinarians are faced with significant conflicts of interest when issuing certificates for the transport and slaughter of acutely and chronically injured livestock. Among others, veterinarians have a duty to minimise pain to the animal, to attend to needs of the farmer and to uphold public health.

Within a wider research project on the ethical challenges facing the veterinary profession in Ireland, this is the third in a series of case studies exploring key issues identified in a recent Policy Delphi consultation process [8]. The other two case studies investigate clinical veterinary services [9] and the on-farm use of veterinary antimicrobials [10]. In this case study, we aim to provide a value-based reflection on the constraints and possible opportunities to best practice emergency and casualty slaughter certification.

## Methods

### Focus groups

A research workshop to explore the constraints and potential opportunities for responsible ECS certification in Ireland was held on 18 June 2015. Thirteen stakeholders agreed to participate in two consecutive focus group sessions (105 and 95 min duration). Purposive sampling of participants was used in order to reflect the range of roles and opinions regarding ECS certification. Selection criteria included seniority, experience with research topic and an active role with a relevant veterinary organisation. Stakeholders included a representative from the regulatory body, local authority veterinarians with research experience in ECS, an animal welfare research scientist, official veterinarians from the competent authority, a private veterinary practitioner and a member of a farming organisation (Table 1). Several participants held more than one active role.

**Table 1:** Participants in focus groups regarding emergency and casualty slaughter (ECS)

	Gender	Stakeholder
ECS-1	M	Veterinary Inspector (Local Authority)
ECS-2	M	Irish Farmers’ Association
ECS-3	F	Veterinary Officers Association
ECS-4	F	Veterinary Inspector (Department of Agriculture, Food and the Marine)
ECS-5	F	Veterinary Inspector (Department of Agriculture, Food and the Marine)
ECS-6	F	Teagasc (Irish Agriculture and Food Development Authority)
ECS-7	M	Farm Animal Welfare Advisory Council
ECS-8	M	Veterinary Inspector (Department of Agriculture, Food and the Marine)
ECS-9	M	Veterinary Inspector (Department of Agriculture, Food and the Marine)
ECS-10	M	Veterinary Council of Ireland
ECS-11	M	Farm Animal Veterinary Practitioner
ECS-12	M	Animal Welfare Research Officer
ECS-13	M	Private Veterinary Practitioner

The sessions were moderated by the last author (AJH) and audio-recorded for qualitative analysis. An interview guide had been developed by the first author (MMS), discussed with co-authors, and revised until a final agreement was reached. A semi-structured approach was used to guide the conversation towards the research questions. In the morning session, each participant was asked to list the three main challenges associated with ECS, and to share their views with the group. This was followed by the appraisal of a comment that had been published several weeks previously in the *Irish Farmers Journal* (16 May 2015), describing a real case scenario of on-farm emergency slaughter, and of a vignette, validated elsewhere [11], describing an ethical dilemma of slaughtering injured livestock (Table 2). The afternoon session was structured around possible strategies that could be adopted to improve ECS, and disagreements and commonalities were explored. After the event, a summary with the main conclusions was sent to participants for comment and clarification.

**Table 2:** Vignette, used in focus group session, describing a case scenario on emergency and casualty slaughter certification

Charlie works as a Temporary Veterinary Inspector (TVI) at a local slaughterhouse in Co. Clare. While on ante-mortem inspection duty, a cull cow arrives with a broken pelvis and he turns a blind eye. “it would be worse to turn her away and isn’t she just about to be put out of her misery anyway?”	

### Data handling and analysis

The sessions were transcribed verbatim, anonymised and a combined deductive and inductive approach to data analysis was applied. As an initial deductive step, the research questions were used to sort and categorise the data according to two thematic, predetermined areas (i.e. challenges and opportunities). An inductive approach was then applied through the use of thematic networks, a commonly used tool for qualitative data analysis [12], particularly in the health sciences [13].

Three stages of analysis were used. Following initial familiarisation with the sorted data, descriptive basic codes to the text units were applied, identifying areas of consensus and conflict within the data, in line with recommendations from Kidd and Parshall [14]. Secondly, the list of basic codes were reviewed and grouped into organising categories, to reflect an emerging pattern. Finally, to form global or macro themes, the list of organising categories were reviewed. This final stage of analysis sought to arrive at conclusive, overarching interpretations of the data, bringing together organising themes to form thematic, analytical networks, which in turn, form the basis of results. Two researchers were involved: preliminary analysis was conducted by a social scientist, independent of the study, followed by a validation analysis performed by the first author (MMS).

## Results

### Challenges

Two overarching themes were identified as key challenges to ECS: dilemmas and conflicts with the commitment to animal welfare, and gaps in governance and support.

- Dilemmas and conflicts with the commitment to animal welfare
  i) Prioritising animal welfare vs. commercial/resource value

There is a conflict between the responsibility of private veterinary practitioners (PVPs) to safeguard the welfare of acutely injured bovines on-farm and the clients’ interest to recover the commercial value of the animal, as illustrated in the following quotes:

*ECS-4: The fact that (…) you have an animal that, from a welfare point of view, the farmer probably should put it down on the farm (…) but he needs to get back its commercial value. [The] big issue is deciding, from the animal’s perspective, from a welfare point of view, what is the best decision to make.*

*ECS-11: (…) the welfare of the farmer, it is a major thing welfare-wise, stress-wise for farmers to lose something that is worth a lot of money and it looks like a lot of waste, and at the other end of the welfare of the farmer, the on-farm slaughter isn’t available around the country.*

Among PVPs, there seem to be concerns that the financial loss for the client - if the animal is sent to the knackery service as opposed to entering the food chain – may trigger the potential loss of clients to other local PVPs who may be willing to certify an acutely injured animal as fit for transportation and fit for human consumption. As a consequence, some PVPs may feel under the “*emotional and financial pressure from the farmer”* [to certify] (ECS-12). With regard to Official Veterinarians (OVs, also called veterinary inspectors), conflicts of interest may arise “*from the top down as well as from their colleagues”* (ECS-6). At the slaughterhouse, it was mentioned that senior line managers, farmers and factory owners are often “*putting pressure on the veterinary inspectors in the plants not to condemn animals on arrival when maybe they should”* (ECS-6). On the other hand, OVs and PVPs may not agree on the clinical interpretations being made on fitness for transport, and this creates additional conflict.

Some participants also highlighted the ethical implications of generating wastage of food and the resource value of the animal. In this regard, there is a “*conflict between a potentially valuable source of meat, (…) and all the resources that went into producing that meat,* versus *animal welfare” (ECS-6).*

ii) Following best judgement vs. following the law

This sub-theme encompasses participants’ opinions regarding the effectiveness of the legislative context in facilitating specific on-farm situations, and in allowing for the best possible solution, as determined by the PVP. There was a consensus among the majority of participants that it is difficult, and often problematic, to work within the confines of the current legislative framework. Others remind the group of the need to prioritise the welfare of the animal at all times, even if in breach of the law:

*The factory is 5 miles down the road but, in my opinion, it is better for the welfare of this animal to be transported and get slaughtered. [However], to the letter of the regulation 1 2005* [6]*, that animals shouldn't be transported. So* [the vet] *could stand in front of the veterinary council for doing what in his pragmatic decision making process is the sensible thing to do in that scenario.* (ESC-13)

This sub-theme overlaps and is related to the wider discussion on the decision-making concerning fitness to transport, and the mediating factors that inform this decision – distance of travel, and the type/extent of the injury and suffering endured by the animal.

iii) Fitness for transport decision-making

This sub-theme encompasses discussion on the challenges around the decision to certify the transportation of an injured animal to the abattoir. A number of participants note the role of risk assessment, experience, and other mediating factors in informing their decision to allow/disallow the transportation of an injured animal. However, there are contrasting opinions, with some participants arguing that, in general, PVPs are reluctant to sign certificates of transport, while others pointing to a “*culture of certifying animals*” as fit for transport.

Confusion is expressed pertaining to the legislative context and definition of fitness to travel, no doubt adding to the dilemma of whether to follow best judgement or the legal context when making the decision regarding certification. Guidelines are called for, to help bring about clarity on the legal requirements for fitness to transport and fitness for human consumption.

*Now who decides whether it’s fit for the journey? (…) “Animals that are injured or that present physiological weaknesses or pathological processes should not be considered fit for transport, and in particular if they are unable to move independently without pain or to walk unaided.”* [6] *So if a cow is lame (…), should that animal be transported?* (ESC-13)

*PVPs find it very hard to know what can be certified. Is it acceptable to certify an animal fit for human consumption with an open fracture?* (ECS-9)

The type or extent of injury and suffering endured by the animal and the distance, or length, of journey to factory facilities are identified as mediating factors in the decision to transport an acutely injured or sick animal, as illustrated in the following example:

*(…) you said I'm happy that this animal is transported a short distance, 20km. Unfortunately the animal is in terrible pain [and] while I think that maybe he could make the 20km (…) the nearest factory is 100km and there is no way that animal would survive that journey. So I can’t sign the certificate on that basis.* (ES-5)

b) Gaps in governance and support

This theme addresses the wider contextual challenges, which in turn influence on-farm decision making. The lack of availability of on-farm slaughter was highlighted which is associated with poor acceptance of emergency slaughter carcasses at the processing plant. It was suggested that *“food business operators are not accepting emergency slaughtered animals because they think it’s counterproductive for the business”* (ECS-9)*.* Although most argue that processors need to take emergency slaughtered animals, and have a social responsibility to do so, others expressed concern that factories may become a means of disposing of sick or injured animals, and should not accept animals that are injured or possibly sick.

Participant observations point to concerns regarding failure to report the actual intake of casualty animals, the non-reporting of frequently occurring cases of injuries, and inconsistencies in approach around the country, and within plants, resulting in confusion among clients. The situation in Northern Ireland was referred to, pointing towards possible measures that may improve governance and support, such as post-mortem reports that increase transparency and accountability.

### Opportunities

Four main themes emerged as possible strategies to address current challenges with ECS. Generally, it was felt that addressing these wider contextual issues would reduce the risk of dilemmas emerging on-farm.

- Training and guidelines
  Participants acknowledged the importance of adequate training and improved guidance to help PVPs dealing with ECS. This includes the regulatory context of on-farm emergency and casualty slaughter, and the practical interpretation of fitness to transport.
- On-farm slaughter availability
  It was felt that equipping slaughterhouses with mobile slaughtering units would provide a practical, humane and economically sound solution to most cases of on-farm emergency slaughter. The existence of nationwide facilities for the latter would alleviate conflicts and dilemmas concerning the need to prioritise animal welfare and concerns pertaining to loss of commercial value. This requires a joint effort between all stakeholders, to improve current guidelines and regulation.
- Incentivisation of best practices
  It was suggested that, in the absence of nationwide availability and acceptance of on-farm emergency slaughter by Food Business Operators (FBOs), consideration should be given to methods to encourage producers to prioritise animal welfare when in conflict with the commercial value of the animal. For example, either subsidising the cost of disposal through the knackery service or implementing a financial penalty through cross-compliance measures. Another suggestion included adopting periods of validity (both in terms of time and distance) for certificates of transportation that may prevent the delay of slaughter or the long distance transportation of animals:
*I would say what you should put on is a period of validity of your cert. You should be putting on there he can be transported 100 miles within the next five hours otherwise the cert is invalid and the animal has to be destroyed* (ECS-1)
- Engagement, communication and consultation
  Engagement between relevant stakeholders, namely the Department of Agriculture, Food and the Marine, and Meat Industry Ireland, to establish the necessary framework to support on-farm slaughter was deemed needed. In addition, reluctance in accepting these animals may arise because *“some of the OVs perhaps are reluctant to accept these because they feel possibly that they can’t stand over how healthy that animal actually was when it was alive”* (ECS-4). It is argued, however, that increased communication would bridge this gap since “*if an OV and a PVP talk[ed] to each other and discuss[ed] the case, nine times out of ten it will be solved.”* (ECS-9)

## Discussion

By relying on a qualitative focus group approach, the purpose of the current case study was to provide a value-based reflection on the challenges associated with emergency and casualty slaughter certification, and to explore possible opportunities for solutions to be developed. Faced with the value-based decision to certify the transport and slaughter of an acutely injured or ill livestock, veterinarians need to consider the range of stakeholders that may be affected, and their often conflicting interests [8, 15].

Multiple barriers to good practice with the slaughter of acutely injured bovines have been identified here and elsewhere [2, 3]. Conflicts and dilemmas arise due to commercial concerns and the gap between governance and provision to facilitate on-farm emergency slaughter. The transport of otherwise healthy livestock that have suffered an accident for casualty slaughter was a key concern during the focus group, because of the implications for animal welfare (i.e. unnecessary pain and suffering caused by transportation to an abattoir) and legal ramifications for the veterinary profession of certifying an acutely injured animal as fit for transport. The conflict arises primarily due to the commercial concerns of the farmer to recover the production costs. Without OFES, and to be legally compliant, acutely injured livestock would either have to be dispatched by the knackery service, or undergo veterinary treatment until the animal is deemed fit for transport, both increasing the financial burden on the farmer.

This study has primarily focused on the role of PVPs in certifying animals for casualty or emergency slaughter but the role of the farmer should be highlighted. Farmers, as the original FBO, have responsibility for the welfare of animals under their care and for the accuracy of supply food chain information accompanying their animals to slaughter. However, decision-making is a shared responsibility of farmers and their PVP and the current lack of OFES puts the farmer in the invidious position of having to choose between animal welfare and financial sustainability and consequently places unreasonable demands on the veterinary professionals. Farmers decisions should be guided by professional advice, however, in some instances farmers may be unaware of the role of the assisting PVP as a technical adviser [16].

A lack of OFES availability is a major obstacle and central to why casualty slaughter predominates. Cullinane and colleagues investigated bovine casualty slaughter at four large abattoirs in the Republic of Ireland [3, 17] and reported that OFES would have been appropriate for 60% of bovines certified for casualty slaughter [3]. More recent research has shown some improvement in the provision of OFES between 2011 and 2013, but casualty slaughter continued to predominate during this period [2]. The study by McDermott and McKevitt revealed that only 10 and 3% of DAFM and Local Authority abattoirs, respectively, accepted OFES. Inconsistencies in the acceptance criteria by OVs and FBOs, and a wide variation in the geographical availability were also reported [2].

Communication, engagement and consultation between stakeholders were considered by study participants as important ways forward to improve the uptake of OFES. In 2015, the European Commission conducted a special Eurobarometer on ‘Attitudes of Europeans towards Animal Welfare’ in 28 Member States. It reported that 94% of participants believed that is important to protect the welfare of farmed animals and 82% wanted better protection for farm animals [18]. In this context, FBOs are doing a disservice to consumers by not providing OFES.

Standardising and providing consistency in OFES also needs to be addressed in guidelines and regulations. Whilst progress has been achieved through amendments to the legal framework to facilitate OFES and the sale of OFES products in the EU, in practice it continues to fail due to the concerns of FBOs. McDermott and McKevitt reported that 89% of FBOs in the Republic of Ireland did not accept OFES, mainly due to a potential negative impact on consumer perception of their business (61%). In addition, OVs were concerned with food safety risks associated with OFES [2]. To increase OFES availability, a food safety analysis is needed to inform decision-making by OVs, PVPs and FBOs.

Incentivising best practice was also considered important by participants, in the move towards OFES. Compliance inspections were identified as one method to reduce inappropriate certification of acutely injured bovines for transportation. Similarly, veterinary participants in a Policy Delphi study also indicated that challenges pertaining to ‘Casualty Slaughter Certification’ would require stringent legal measures, namely compliance inspections and improved legislation/regulations [8]. Conversely, subsidising the disposal costs of acutely injured bovines through the knackery service would help to reduce the commercial losses for the producer. This mechanism has previously been used by DAFM, referred to as the Fallen Animal Scheme, which ended in 2009. Finally, the adoption of periods of validity for certificates of transportation was also suggested at the workshop. A similar suggestion had been made by Cullinane and colleagues to prevent casualty animals from being slaughtered several days after being transported [17].

In some cases, legislation was considered to present a barrier to best judgement by the veterinarian. ‘Emergency killing’ and ‘casualty slaughter’ are common terms in the farming industry, and often used interchangeably. European regulations do not attempt to differentiate the two, thus adding to the confusion. Moreover, regulatory provisions exist defining what is meant by ‘fitness for transport’, namely that animals shall not be considered fit for transport if they are unable to move independently without pain or to walk unassisted [6]. However, the present research suggests that conflicts can arise between PVPs and OVs, and between these veterinarians and other stakeholders, regarding the interpretation and decision-making of fitness for transport.

Looking at examples from other jurisdictions, it can be argued that confusion regarding the regulatory landscape and the best course of action is not exclusive to Irish veterinarians. In effect, an exploratory study of the ethical challenges faced by Austrian veterinary officers has shown that “conflicting norms and values are the rule in veterinary officers’ daily job – not the exception. They are essential and unavoidable and not the result of a lack of individual competence, conscience or personal skills” ([19], p. 287). From a UK perspective, the challenges facing OVs working in post-Brexit Britain, and their role in preventing animal cruelty and increasing efficiency and safety of meat inspection, have recently been emphasised [20].

Moreover, participants in the present study highlighted the role of client pressure on clinical decision-making by the PVP but also pressure faced by OVs from a number of stakeholders. Such competing interests could mean that the values driving PVPs and OVs decision-making do not necessarily overlap. This represents a potential area of conflict between PVPs and OVs, one that can impact negatively on the reputation of the veterinary profession and the welfare of animals.

Focus group participants identified training of PVPs as an opportunity to improve the provision of OFES. This has also been mirrored by a survey of PVPs in Ireland [2]. The transition to OFES requires behavioural change and factors such as continuing veterinary education in ethics should be part of this strategy. Following the four-part conceptual model of veterinary ethics teaching, training should include the relevant norms and regulations, topics on animal welfare, ethical theories and professionalism [21]. Education in ethics can help veterinarians recognise the values and viewpoints of others, to develop value-aware communication skills as well as informed decision-making skills [22, 23].

The present case study is part of a wider workshop where participants were divided into smaller groups, on the grounds of their expertise, and some limitations should be acknowledged. This investigation relied on two focus group sessions and on the same group for both sessions. Nonetheless, the group was sufficiently diverse in order to minimise a cohort effect. In fact, several participants had more than one professional role. Further, it was the role of the moderator to ensure that every participant had a chance to meaningfully contribute to the debate. The debate was centred on bovine animals, leaving welfare concerns with other production animals, such as pigs and sheep, largely unexplored. This bias may reflect the greater economic value of bovines in Ireland, compared to other livestock species. Results from this study should be extrapolated with caution since the small number of participants involved in this study is unlikely to represent the full range of views of every stakeholder involved with ECS in Ireland.

## Conclusion

Four main strategies emerged from the focus group discussion to address current challenges with emergency and casualty slaughter.

1. Support should be given to the nationwide availability and acceptance of on-farm emergency slaughter. There needs to be engagement, communication and consultation between all stakeholders (e.g. Veterinary Ireland, Department of Agriculture, Food & the Marine, Meat Industry Ireland, Irish Farmers Association, FBOs) to improve current guidelines and regulation.
2. Training and guidelines for PVPs on the regulatory context of on-farm emergency slaughter and casualty slaughter, on the interpretation of fitness to transport, effective communication and ethical decision-making should become available.
3. In the absence of nationwide availability and acceptance of on-farm emergency slaughter by FBOs, consideration should be given to methods to encourage all those involved in the food chain to prioritise animal welfare when it is in conflict with the commercial value of the animal. For example, either subsidising the cost of disposal through the knackery service or implementing a financial penalty through cross compliance measures.
4. Within a climate of recovery for the Irish economy, DAFM should consider the interim re-introduction of the fallen animal scheme as a public good to support farm animal welfare.

## Abbreviations

ECS: Emergency and casualty slaughter
FAWAC: Farm Animal Welfare Advisory Council
FBOs: Food business operators
OFES: On farm emergency slaughter
OVs: Official veterinarians
PVPs: Private veterinary practitioners
